# Impact of delivery mode on childbirth satisfaction, birth memory, and pregnancy avoidance

**DOI:** 10.1186/s12884-025-07887-4

**Published:** 2025-09-30

**Authors:** Gizem Çitak, Demet Çakir, Serpil Toker

**Affiliations:** 1 Midwifery Department, Doctor Faculty Member, Tokat Gaziosmanpaşa University, Faculty of Health Sciences, Tokat, Turkey; 2 Midwifery Department, Lecturer, Tokat Gaziosmanpaşa University, Faculty of Health Science, Tokat, Turkey

**Keywords:** Birth memory, Childbirth satisfaction, Mode of delivery, Pregnancy avoidance

## Abstract

**Background:**

Women’s perceptions of childbirth can be influenced by individual differences, previous birth experiences, and the social-medical context. However, there is limited research on how the mode of delivery affects childbirth satisfaction, pregnancy avoidance, and birth memory. This study aims to investigate the impact of vaginal and cesarean deliveries on these factors.

**Methods:**

This descriptive, comparative and cross-sectional study was conducted with 486 postpartum women (243 vaginal and 243 cesarean deliveries) in a maternity ward. The Personal Information Form and the Birth Satisfaction Scale (BSS-R) were used in the first interview at 12 h postpartum. In the second interview on postpartum day 7, the Desire to Avoid Pregnancy Scale (DAPS) and the Birth Memory and Recall Scale (BMRS) were used. Data were analyzed using descriptive statistical methods, Independent Sample T-test, Chi-Square, One-Way ANOVA, Two-Way ANOVA, and regression analysis.

**Results:**

There is a significant difference between the groups in terms of the total BSS-R score. In addition, there are significant intergroup differences in the sub-dimensions of memory centrality and consistency from the BMRS sub-dimensions (*p* < 0.05).

**Conclusion:**

It was observed that women who gave birth by cesarean section generally reported higher satisfaction scores, but they showed a higher tendency to avoid pregnancy. In contrast, birth memory and recall characteristics were more pronounced in women who gave birth vaginally. However, there was no significant difference between the groups in terms of the total score of the BMRS. Differences were limited to some sub-dimensions only.

## Introduction

Providing women with accurate information from trusted sources and guiding them to the mode of delivery that best suits their expectations, wishes and clinical circumstances is crucial for optimal maternal and infant health outcomes. In the existing literature, there are different findings regarding the effects of vaginal and cesarean deliveries on mother-infant interactions. Some studies suggest that cesarean deliveries may be associated with adverse outcomes such as maternal pain, fatigue, and delayed milk production, whereas vaginal deliveries are generally reported to result in less pain and earlier milk production. However, more research is needed to determine whether these associations are specific to mode of delivery or associated with other factors. Birth satisfaction continues to be an important determinant of women’s perception of the birth experience [1, 2].

Women who report positive birth experiences tend to have higher self-confidence and stronger bonding with their infants, contributing to better maternal mental health and well-being [3]. In contrast, negative birth experiences are often linked to a variety of challenges, such as breastfeeding difficulties, impaired maternal bonding, postpartum depression, neglect of infant care, sexual dysfunction, and difficulties in deciding whether to have another child [4, 5]. Additionally, women who have had negative childbirth experiences may develop a fear of future pregnancies, which can influence their desire to avoid them [6].

Perceptions of childbirth are highly individualized, shaped not only by personal experiences but also by the meanings attributed to those experiences. Societal, cultural, and contextual factors further influence how women perceive childbirth [7]. A key aspect of these perceptions is *birth memory*, which refers to the recollection of childbirth experiences stored in long-term memory. Studies on memory and recall suggest that childbirth events occupy a significant place in a woman’s life, often affecting her long-term psychological well-being. These memories can have a lasting impact, influencing how women view future pregnancies and childbirths [8].

Although there are various assumptions about the effect of birth memory on future pregnancy perspectives in the existing literature, there is a need for high-quality, long-term data on how this relationship is shaped. In particular, the effects of different modes of birth and birth experiences on this memory have not been fully elucidated. At this point, our study contributes to a better understanding of the postpartum psychological process by examining the relationship between birth memory and birth satisfaction. It also aims to fill the gaps in the literature on how birth memory may affect women’s attitudes and behaviors towards their subsequent pregnancies. This research aims to provide insights into the psychological and emotional dimensions of childbirth and thus contribute to better maternity care practices and informed health care decisions.

## Materials and methods

### Population and sample of the study

The study was conducted among women in the puerperium hospitalized in the obstetrics ward of a university hospital in the Central Black Sea region between 01/09/2023, and 01/09/2024. A non-probability sampling method was employed, and the sample size was determined using G*Power 3.1.9.7 software [9], with an effect size of f² = 0.02, based on Cohen’s (1988) recommendation for a small effect size [10, 11], a confidence level of 80%, and a margin of error of 5%. Consequently, the sample size was calculated as 486 postpartum women, equally divided between 243 vaginal deliveries and 243 cesarean deliveries. Inclusion criteria consisted of newly delivered puerperium women who were either primiparous or multiparous, and who voluntarily agreed to participate, with informed consent obtained. Exclusion criteria included asylum seekers or refugees, participants who decided to withdraw after enrolling, and those with incomplete or missing data forms.

### Data collection tools

Data were collected using the following instruments: the Personal Information Form, the Childbirth Satisfaction Scale (BSS-R), the Desire to Avoid Pregnancy Scale (DAPS), and the Birth Memory and Recall Scale (BMRS).

#### Personal information form

Developed by the researchers based on a literature review, this form includes 17 questions about the sociodemographic and obstetric characteristics of postpartum women [12, 13].

#### Childbirth satisfaction scale (BSS-R)

Developed by Gökmen et al. [14], the BSS-R is a 10-item scale with three subdimensions: Quality of Care, Stress Experienced during Labor, and Personal Characteristics of the Woman. It uses a 5-point Likert scale (0 = Strongly disagree, to 4 = Strongly agree) and is administered within the first ten days postpartum [14]. The total score ranges from 0 to 40, with higher scores indicating greater satisfaction. Four items [2, 4, 7, 8] are reverse-scored. The scale’s Cronbach’s alpha, reported as 0.72 was found to be 0.91 in this study (Table 3).

#### Desire to avoid pregnancy scale (DAPS)

It is a psychometric scale developed to assess individuals' desire to avoid pregnancy; that is, their level of not wanting a pregnancy and their intellectual, emotional, and expectational attitudes towards it. Özten ve Bilgin [15] validated DAPS, which consists of 14 items measuring three factors: Cognitive Desires and Preferences, Affective Feelings and Attitudes, and Expected Objective Results. It uses a 5-point Likert scale (0 = Strongly agree to 4 = Strongly disagree), with higher scores reflecting a stronger desire to avoid pregnancy [15]. Cronbach’s alpha values reported for the scale range from 0.90 to 0.95. In this study, it was 0.75 (Table 3).

#### Birth memory and recall scale (BMRS)

Developed by Topkara and Çağan (2021), BMRS includes 21 items across six subdimensions: Emotional Memory, Centrality of Memory, Consistency, Reliving, Sensory Memory, and Recall. Each subdimension is scored separately, with higher scores indicating more positive birth memories. Items 1, 3, and 11 are reverse-scored [16]. The scale’s Cronbach’s alpha is 0.80; however, in this study, it was 0.75 (Table 3).

### Implementation of the study

The purpose and methodology of the study were explained to postpartum women admitted to the Obstetrics and Gynecology Service, and informed consent was obtained. According to hospital procedures, women are admitted to the Obstetrics and Gynecology Service for follow-up and care after delivery; women who give birth vaginally are discharged after one day and women who give birth by cesarean section are discharged after two to three days. In our study, the first interview was conducted face-to-face at the 12th hour postpartum. The Personal Information Form, and Birth Satisfaction Scale were administered to women who agreed to participate in the study.

**Fig. 1:**
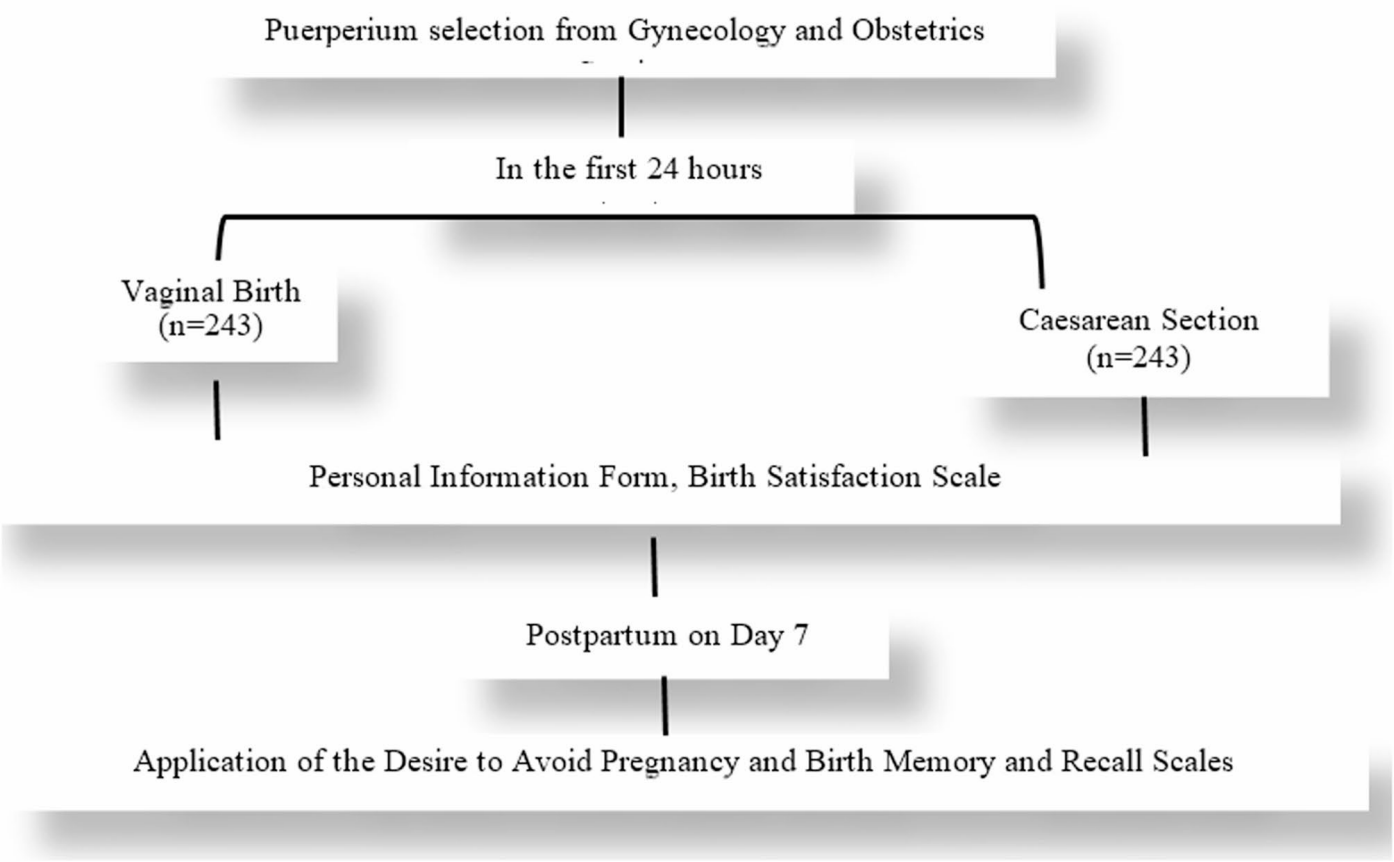
Implementation flowchart

According to the hospital protocol, women are called to the hospital for routine follow-up one week after delivery. In accordance with this protocol, women were called back to the Obstetrics and Gynecology Outpatient Clinics for follow-up one week after delivery, and the Pregnancy Avoidance Desire Scale and Birth Memory and Recall Scale were administered face-to-face. The researchers ensured that the data collection forms were filled in before collecting them. This process took approximately 10–15 min (Fig. 1).

### The ethical aspect of the study

Approval was obtained from the Non-Interventional Clinical Research Ethics Committee of a university in the Middle Black Sea region (Decision No: 203/15, Decision Date: 31.08.2023). Additionally, institutional permission was granted by the hospital where the study was conducted (Decision No: 449097, Decision Date: 10.07.2024). The study was conducted in compliance with the rules of the Helsinki Declaration and informed consent was obtained from the postpartum women who agreed to participate in the study.

### Statistical analysis

The statistical analysis of the obtained data was performed using the SPSS 22.0 software. Descriptive statistical measures (mean, standard deviation, minimum and maximum values, and percentages) were used. Since the assumptions for parametric tests were met, the differences between the means of two independent groups were analyzed using an independent samples t-test. For more than two independent groups, one-way analysis of variance (ANOVA) was conducted with Tukey’s post hoc test. To determine the differences between categorical data, the Chi-square test was used, with the Bonferroni-corrected Z test for post hoc analysis. A two-way ANOVA was applied to compare variables across vaginal and cesarean birth groups, and multiple linear regression was used to determine the variables affecting vaginal and cesarean birth outcomes. The significance level was set at 0.05.

While evaluating the normality analysis of the data, Kolmogorov-Smirnov test statistics, p value, skewness, and kurtosis coefficients were examined to determine which distribution the data of the variables came from. In line with Tabachnick and Fidell’s (2013) recommendation, it was accepted that the distribution of the data was within normal limits if the p value was greater than 0.05 or the skewness and kurtosis coefficients were within ± 2 limits.

## Results

Sociodemographic characteristics of the pregnant women are given in Table 1. The mean age of women who delivered vaginally was 27.56 ± 5.80 years. The mean age of women who delivered by cesarean section was 29.52 ± 6.03 years. A statistically significant difference was found between the groups in terms of age range and mean age (*p* < 0.05).

**Table 1:** Distribution of Sociodemographic Characteristics by Type of Birth and Between-Group Comparison (*N*=486)

**Features**	**Vaginal delivery (** ***n*** **=243)**		**Caesarean section (** ***n*** **=243)**		**Test value/p**
	**n**	**%**	**n**	**%**	
**Age**					
18-28 age	137	56,4a	113	46,5b	***χ2= 4,745***
29 years and above	106	43,6a	130	53,5b	***p=0,029***
**Mean age**					***t=-3,642***
**X̄±SS (min-max)**	27,56±5,80 (18-45)		29,52±6,03 (18-45)		***p<0,001***
**Education status**					
Literate	12	4,9	7	2,9	χ2= 2,193 p=0,533
Primary education	137	56,4	137	56,4	
High school	64	26,4	73	30,0	
University and above	30	12,3	26	10,7	
**Partner's education status**					
Literate	8	3,3	3	1,2	χ2= 3,043 p=0,385
Primary education	131	53,9	143	58,9	
High school	77	31,7	72	29,6	
University and above	27	11,1	25	10,3	
**Relationship status with partner**					
Yes	127	52,3	128	52,7	χ2= 0,008
No	116	47,7	115	47,3	p=0,928
**Family type**					
Nuclear Family	214	88,1	220	90,5	χ2= 0,775
Extended Family	29	11,9	23	9,5	p=0,379
**TOTAL**	**243**	**100,0**	**243**	**100,0**	

χ2: chi-square test, t: Independet sample t test, X̄: mean value, *SD *Standard Deviation

Obstetric characteristics of pregnant women are given in Table 2. It was observed that 53.9% of the women who had vaginal delivery were primiparous. The mean number of children planned by their families was 3.15 ± 1.26. The mean number of children planned by their families was 3.14 ± 1.12. A significant difference was found between the groups in terms of previous delivery method (*p* < 0.05) (Table 2).

**Table 2:** Distribution of Obstetric Characteristics According to Delivery Type and Inter-Group Comparison (*N*=486)

**Features**	**Vaginal delivery (** ***n*** **=243)**		**Caesarean section (** ***n*** **=243)**		**Test value/p**
	**n**	**%**	**n**	**%**	
**Number of pregnancy**					
Primipar	131	53,9	125	51,4	χ2= 0,297
Multipar	112	46,1	118	48,6	p=0,586
**Presence of abortion**					
Yes	47	19,3	56	23,0	χ2= 0,998
No	196	80,7	187	77,0	p=0,318
**Presence of curettage**					
Yes	27	11,1	29	11,9	χ2= 0,081
No	216	88,9	214	88,1	p=0,776
**Previous type of birth (n=245)**					
Vaginal delivery	107	91,5^a^	10	7,8^b^	***χ2= 171,395***
Caesarean section	10	8,5^a^	118	92,2^b^	***p<0,001***
**Previous use of family planning methods**					
Yes	169	69,8	187	77,0	χ2= 3,148
No	73	30,2	56	23,0	p=0,076
**Number of children planned for the family**					
Child	16	6,6	11	4,5	χ2= 0,981 p=0,612
2-3 children	142	58,4	145	59,7	
4 children and above	85	35,0	87	35,8	
**Average number of children planned for the family** **X̄±SS (min-max)**	3,15±1,26 (1-7)		3,14±1,12 (1-7)		t=0,114 p=0,909
**Baby's gender**					
Girl	125	51,4	122	50,2	χ2= 0,074 p=0,785
Boy	118	48,6	121	49,8	
**TOTAL**	**243**	**100,0**	**243**	**100,0**	

χ2=chi-square test, t: Independet sample t test, X̄: mean value, *SD *Standard Deviation

The mean total score for the BSS-R among women who had a vaginal delivery was 21.01 ± 4.32. The subscale means were as follows: quality of care: 10.89 ± 3.41; stress experienced during labor, 7.13 ± 2.60; and women’s personal characteristics, 2.98 ± 1.86. The mean total score for the DAPS was 2.29 ± 0.61. The subscale means were as follows: cognitive desires and preferences, 2.29 ± 0.57; emotional feelings and attitudes, 2.26 ± 0.89; and expected objective outcomes, 2.29 ± 0.88.

The mean total score for the BMRS was 4.54 ± 0.69. The subscale means were as follows: emotional memory, 4.10 ± 0.88; centrality of memory, 4.65 ± 1.11; coherence, 4.38 ± 1.22; reliving, 4.84 ± 1.24; sensory memory, 4.61 ± 1.17; and recall, 4.83 ± 1.35 (Table 3).

**Table 3:** Distribution of the total and sub-dimensions of the Birth Satisfaction Scale, Pregnancy Avoidance Desire Scale, and Birth Memory and Recall Scale by birth type, and the comparison between the groups

**Scales**	**Vaginal delivery (** ***n*** **=243)**	**Caesarean section (** ***n*** **=243)**	**Cronbach alpha**	**p** ^a^
	**X̄** **±SD** ** (min-max)**	**X̄** **±SD** ** (min-max)**		
**Birth Satisfaction Scale**	21,01±4,32 (8-38)	21,84±3,84 (9-32)	0,91	***0,026***
Quality of care	10,89±3,41 (1-16)	11,44±3,79 (2-16)	0,80	0,098
Stress experienced during labor	7,13±2,60 (0-15)	7,42±2,44 (0-16)	0,73	0,202
The woman's personal characteristics	2,98±1,86 (0-8)	2,97±1,78 (0-8)	0,92	0,980
**Desire to Avoid Pregnancy Scale **	2,29±0,61 (0,36-4)	2,33±0,53 (1,29-4)	0,75	0,411
Cognitive desires and preferences	2,29±0,57 (0-4)	2,34±0,52 (1,13-4)	0,73	0,378
Affective feelings and attitudes	2,26±0,89 (0-4)	2,31±0,85 (0,33-4)	0,79	0,546
Expected objective results	2,29±0,88 (0,33-4)	2,33±0,76 (0,67-4)	0,72	0,647
**Birth Memory and Recall Scale**	4,54±0,69 (1,81-6,38)	4,48±0,72 (2-6,48)	0,75	0,357
Emotional memory	4,10±0,88 (1-7)	4,08±0,93 (1,20-7)	0,80	0,819
Centrality of memory	4,65±1,11 (1-7)	4,43±1,25 (1-7)	0,74	***0,049***
Consistency	4,38±1,22 (2-7)	4,65±1,48 (1-7)	0,80	***0,029***
Live again	4,84±1,24 (1-7)	4,80±1,40 (1-7)	0,79	0,720
Sensory memory	4,61±1,17 (1-7)	4,43±1,30 (1-7)	0,75	0,101
Remembering	4,83±1,35 (1-7)	4,87±1,62 (1-7)	0,78	0,785

The mean total score for the BSS-R among women who underwent cesarean delivery was 21.84 ± 3.84. The subscale means were as follows: quality of care: 11.44 ± 3.79; stress experienced during labor: 7.42 ± 2.44; and women’s personal characteristics: 2.97 ± 1.78. The mean total score for the DAPS was 2.33 ± 0.53. The subscale means were as follows: cognitive desires and preferences, 2.34 ± 0.52; emotional feelings and attitudes, 2.31 ± 0.85; and expected objective outcomes, 2.33 ± 0.76.

The mean total score of the Birth Memory and Recall Scale (BMRS) in those who delivered vaginally was 4.54 ± 0.69. The mean subscale scores were as follows: emotional memory: 4.10 ± 0.88; centrality of memory: 4.65 ± 1.11; coherence: 4.38 ± 1.22; reliving: 4.84 ± 1.24; sensory memory: 4.61 ± 1.17; and recall: 4.83 ± 1.35 (Table 3).

In the comparison of the total and sub-dimensional mean scores of the scales according to the type of delivery, it was found that the mean birth satisfaction score of cesarean births was higher than vaginal births, and there was a significant difference between the groups (*p* = 0.026; *p* < 0.05). It was determined that the mean score of the centrality of memory in the BMRS was higher in those who gave birth vaginally, and there was a significant difference between the groups (*p* = 0.049). It was found that the mean score of the consistency sub-dimension of the BMRS was higher in those who gave birth by cesarean section, and there was a significant difference between the groups (*p* = 0.029; *p* < 0.05) (Table 3).

When the reliability levels of the internal validity coefficients of the scales used in the study were examined, it was determined that the overall overall reliability level of the BSS-R was high (cronbach Alpha = 0.091), and the overall reliability level of the DAPS and BSS-R scales was quite reliable (cronbach Alpha = 0.075) (Uzunsakal & Yıldız, 2018) (Table 3).

In the comparison of the mean BSS-R total score with sociodemographic characteristics, no significant difference was found between vaginal delivery and the variables (*p* > 0.05), while a significant difference was found between cesarean delivery and educational status and spousal kinship status (*p* < 0.05). In comparing the BSS-R total score across various sociodemographic characteristics, a significant difference in the type of delivery was found with respect to educational status, spouse’s educational status, and kinship status with spouse (*p* < 0.05). It was found that the mean BSS-R score of university graduates who had cesarean delivery was significantly higher than that of graduates who had vaginal delivery. It was determined that the mean BSS-R score of those who were accompanied by their spouses in cesarean deliveries was significantly higher than that of those who delivered vaginally (Table 4).

**Table 4:** Comparison of BSS-R, DAPS, and BMRS Total Scores Based on Sociodemographic Characteristics by Mode of Delivery (*N*=486)

**Features**	**Birth Satisfaction Scale**			**Desire to Avoid Pregnancy Scale**			**Birth Memory and Recall Scale**		
	**Vaginal delivery** **(n=243)**	**Caesarean section** **(n=243)**	**Test value/p** ^a^	**Vaginal** **Delivery** **(n=243)**	**Caesarean section** **(n=243)**	**Test value/p** ^a^	**Vaginal delivery** **(n=243)**	**Caesarean section** **(n=243)**	**Test value/p** ^a^
	**X̄±SD**	**X̄±SD**		**X̄±SD**	**X̄±SD**		**X̄±SD**	**X̄±SD**	
**Age**			2,315/0,075			0,583/0,626			2,610/0,051
18-28 age	20,76±3,92	21,60±4,02		2,30±0,61	2,29±0,50		4,62±0,65	4,40±0,72	
29 years and above	21,33±4,79	22,06±3,68		2,27±0,62	2,36±0,55		4,43±0,72	4,55±0,71	
**t/p**	-0,999/0,319	-0,930/0,354		0,433/0,666	-0,998/0,319		***2,142/0,033***	-1,575/0,117	
**Education status**									
Literate	22,83±5,06	20,14±5,36	***2,690/0,010***	2,23±0,50	2,09±0,51	***2,334/0,024***	4,83±0,65a	4,40±0,49	***4,998/0,000***
Primary education	20,60±3,61	22,22±3,62a		2,24±0,52a	2,30±0,47		4,65±0,61b	4,62±0,68a	
High school	21,43±4,83	22,02±3,54		2,25±0,60	2,33±0,54		4,45±0,70	4,37±0,74	
University and above	21,26±5,64	19,80±4,74a		2,59±0,89a	2,56±0,74		4,12±0,84ab	4,08±0,73a	
**F/p**	1,358/0,256	***3,500/0,016***		***2,828/0,039***	2,323/0,076		***6,353/0,000***	***5,073/0,002***	
**Partner's education status**			***2,262/0,028***			***2,064/0,046***			***6,087/0,000***
Literate	23,75±3,24	18,00±1,00		2,15±0,43	1,78±0,25		4,63±0,57a	3,96±0,47	
Primary education	20,76±4,01	21,84±3,61		2,23±0,52	2,32±0,50		4,67±0,58b	4,54±0,74a	
High school	21,15±4,59	22,44±3,93		2,30±0,67	2,31±0,49		4,54±0,71c	4,53±0,62b	
University and above	21,03±5,13	20,60±4,61		2,57±0,78	2,50±0,75		3,89±0,80abc	4,06±0,74ab	
**F/p**	1,245/0,294	2,503/0,060		2,541/0,057	1,972/0,119		***10,641/0,000***	***3,819/0,011***	
**Relationship status with partner**									
Yes	20,96±3,83	21,29±3,53	***3,337/0,019***	2,16±0,51	2,21±0,48	***9,019/0,000***	4,68±0,64	4,45±0,66	***3,942/0,009***
No	21,06±4,82	22,46±4,09		2,43±0,67	2,46±0,55		4,39±0,71	4,51±0,78	
**t/p**	-0,179/0,858	***-2,379/0,018***		***-3,430/0,000***	***-3,830/0,000***		***3,357/0,000***	-0,625/0,532	
**Family type**			1,735/0,159			***4,374/0,005***			0,509/0,677
Nuclear Family	21,00±4,44	21,88±3,92		2,33±0,62	2,35±0,54		4,53±0,71	4,49±0,73	
Extended Family	21,06±3,35	21,47±2,98		2,00±0,43	2,11±0,33		4,61±0,44	4,39±0,53	
**t/p**	-0,069/0,945	0,484/0,629		***3,616/0,000***	***3,081/0,004***		-0,576/0,565	0,587/0,558	

*F *One-Way ANOVA, *t *Independent Sample t-test, *X̄ *Mean, *SS *Standard Deviation, ^a^Two-Way ANOVA, a-c: Significant differences exist among groups with the same letter

In comparing the DAPS total score mean with sociodemographic characteristics, a significant difference was identified between vaginal delivery and educational status, spousal kinship status, and family type (*p* < 0.05), as well as between cesarean delivery and spousal kinship status and family type (*p* < 0.05). In the comparison of DAPS total score with sociodemographic characteristics, a significant difference was found between educational status, spouse’s educational status, kinship status with spouse, and family type for the type of delivery variable (*p* < 0.05). It was determined that university graduate women who delivered vaginally avoided pregnancy more than university graduates who delivered by cesarean section. In women who had cesarean delivery, it was found that the mean DAPS scores of those married to their husbands and lived in nuclear families were higher than the scores of those who had vaginal delivery (Table 4).

In the comparison of the mean BMRS total score with sociodemographic characteristics, it was found that there was a significant difference between vaginal delivery and, specifically, age, education status, spouse education status, and kinship status with spouse (*p* < 0.05). Furthermore, there was a significant difference between cesarean delivery and, specifically, education status and spouse education status (*p* < 0.05). In comparing the BMRS total score across sociodemographic characteristics related to the type of delivery, a significant difference was found with educational status, spouse’s educational status, and kinship status with spouse (*p* < 0.05). It was found that the mean BMRS score was significantly higher in women who delivered vaginally, than in women who delivered by cesarean section at all educational levels (Table 4).

In the comparison of the mean BSS-R total score with obstetric characteristics, no significant difference in mean scores was found between vaginal delivery and other variables (*p* > 0.05), while a significant difference was found between cesarean delivery and the presence of abortion (*p* < 0.05). In comparing the total score of BSS-R with obstetric characteristics, a significant difference (*p* < 0.05) was found related to the variable type of delivery and the presence of abortion. It was found that the mean BSS-R score of women who had cesarean delivery was significantly higher than in women who had vaginal delivery, particularly among those who had not had a previous abortion (Table 5).

**Table 5:** Comparison of BSS-R, DAPS, and BMRS Total Scores Based on Obstetric Characteristics According to Delivery Type (*N*=486)

**Features**	**Birth Satisfaction Scale**			**Desire to Avoid Pregnancy Scale**			**Birth Memory and Recall Scale**		
	**Vaginal delivery** **(** ***n*** **=243)**	**Caesarean section** **(** ***n*** **=243)**	**Test value/p** ^a^	**Vaginal** **Delivery** **(** ***n*** **=243)**	**Caesarean section** **(** ***n*** **=243)**	**Test value/p** ^a^	**Vaginal delivery** **(** ***n*** **=243)**	**Caesarean section** **(** ***n*** **=243)**	**Test value/p** ^a^
	**X̄±SD**	**X̄±SD**		**X̄±SD**	**X̄±SD**		**X̄±SD**	**X̄±SD**	
**Number of pregnancy**			1,911/0,127			1,465/0,223			0,645/0,586
Primipar	19,80±5,35	23,30±3,97		2,67±0,45	2,67±0,61		4,74±0,91	4,41±0,64	
Multipar	21,21±4,91	21,98±3,81		2,35±0,65	2,37±0,55		4,49±0,69	4,47±0,76	
	-0,645/0,520	-0,532/0,595		-1,472/0,142	-1,206/0,229		1,047/0,296	0,194/0,847	
**Presence of abortion**			***3,078/0,027***			0,348/0,791			1,820/0,143
Yes	21,34±5,53	20,91±3,81		2,33±0,66	2,32±0,57		4,34±0,66	4,46±0,66	
No	21,02±4,49	23,00±3,60		2,38±0,64	2,45±0,55		4,60±0,70	4,47±0,81	
	0,469/0,641	***-2,093/0,037***		0,551/0,582	-0,167/0,867		***-2,186/0,030***	-0,271/0,787	
**Presence of curettage**			1,741/0,158			0,623/0,600			0,356/0,256
Yes	20,92±6,17	21,51±3,71		2,33±0,67	2,23±0,49		4,27±0,64	4,51±0,63	
No	21,22±4,51	22,25±3,86		2,37±0,65	2,44±0,57		4,57±0,70	4,45±0,78	
	-0,083/0,934	-0,492/0,623		0,351/0,726	-1,104/0,271		***-2,180/0,030***	0,213/0,832	
**Previous type of birth (** ***n*** **=245)**			2,567/0,055			2,567/0,055			1,547/0,203
Vaginal delivery	21,40±4,86	23,30±3,97		2,34±0,62	2,67±0,61		4,54±0,69	4,41±0,64	
Caesarean section	18,50±4,88	21,98±3,81		2,63±0,91	2,37±0,55		4,04±0,54	4,47±0,76	
	1,802/0,074	1,045/0,298		-1,363/0,175	1,601/0,112		***2,219/0,028***	-0,243/0,809	
**Previous use of family planning methods**			2,503/0,059			1,507/0,213			***6,374/0,000***
Yes	20,72±4,83	21,84±3,67		2,36±0,56	2,37±0,54		4,58±0,66	4,54±0,67	
No	21,97±5,04	22,62±4,13		2,36±0,80	2,44±0,60		4,34±0,74	4,31±0,87	
	-1,232/0,219	-0,849/0,398		-1,209/0,229	***-2,073/0,041***		***3,008/0,003***	***2,476/0,015***	
**Number of children planned for the family**									
Child	20,80±3,49	21,00±3,40	1,818/0,108	2,68±0,57	2,51±0,70	1,316/0,256	4,21±1,23	4,31±0,53	2,014/0,075
2-3 children	21,48±4,84	21,81±3,65		2,41±0,77	2,30±0,57		4,53±0,67	4,33±0,69a	
4 children and above	20,80±5,16	22,56±4,08		2,28±0,47	2,50±0,51		4,49±0,66	4,66±0,80a	
	0,919/0,400	1,145/0,320		0,738/0,479	2,451/0,088		0,327/0,721	***4,171/0,017***	
**Baby's gender**			2,451/0,063			0,442/0,723			1,291/0,277
Girl	21,22±4,26	22,65±3,76		2,40±0,66	2,50±0,60		4,63±0,66	4,54±0,75	
Boy	21,07±5,62	21,56±3,83		2,32±0,63	2,30±0,50		4,34±0,70	4,40±0,74	
	-0,120/0,904	1,626/0,105		-0,258/0,796	0,815/0,416		1,449/0,149	0,985/0,326	

*F* One-Way ANOVA, *t *Independent Sample t-Test, *X̄ *Mean Value, *SD *Standard Deviation, ^a^Two-Way ANOVA, a-c: Significant differences exist between groups sharing the same letter

The comparison of the mean total score of DAPS and obstetric characteristics revealed no significant difference between vaginal delivery and obstetric characteristics (*p* > 0.05). However, there was a significant difference between cesarean delivery and the previous use of a family planning method (*p* < 0.05). It was determined that the mean DAPS score of women who had previously used family planning and underwent cesarean delivery was significantly higher than those of women who underwent vaginal delivery. In comparing the DAPS total score with obstetric characteristics related to the type of delivery variable, it was determined that there was no significant difference between the variables (*p* > 0.05) (Table 5).

In the comparison of the mean BMRS total score with obstetric characteristics, a significant difference was found between vaginal delivery and factors such as the presence of abortion, presence of curettage, previous mode of delivery, and previous use of a family planning method (*p* < 0.05). Additionally, there was a significant difference between cesarean section, and previous use of a family planning method, and the number of children planned for the family (*p* < 0.05). In the comparison of the total score of BMRS and obstetric characteristics, a significant difference in the type of delivery variable was determined for the use of family planning methods prior to delivery (p <0.05). It was found that the mean BMRS score of women who had previously used family planning methods and gave birth vaginally was significantly higher than that of women who gave birth by cesarean section (Table 5).

In the regression analysis of the variables thought to affect scale scores in vaginal and cesarean deliveries, a statistically significant model was identified between the scales and variables for both birth types (*p* < 0.05). Among women who had a vaginal delivery, kinship with the spouse and family type significantly affected the total score of the DAPS. Kinship with the spouse had a negative effect, while family type had a positive effect. Additionally, the presence of abortion had a positive effect on the total score of the BMRS.

In women who had a cesarean delivery, education level and kinship with the spouse were found to significantly affect the total score of the BSSR. Both education level and kinship with the spouse had a negative effect. Regarding the DAPS total score, kinship with the spouse and previous use of a family planning method were found to be significant factors, both of which positively influenced the total score. For the BMRS total score, education level, previous use of a family planning method, and the number of children planned for the family were significant variables. Education level and prior use of a family planning method had a negative effect, whereas the planned number of children for the family had a positive effect (*p* < 0.05) (Table 6).

**Table 6:** Regression Analysis of Factors Affecting the BSS-R, DAPS and BMRS by Type of Delivery

**DAPS** **Independent Variables**		**β**	**S.E.**	**p** ^a^	**95% CL**		**Model Fit**
					**Lower**	**Upper**	
**VAGINAL DELIVERY**	Educational status	0,032	0,033	0,317	-0,031	0,095	R2 = 0,064
	Kinship with spouse	***0,231***	***0,078***	***0,003***	***0,077***	***0,385***	F=6,510
	Family type	***-0,282***	***0,118***	***0,018***	***-0,515***	***-0,049***	***p<,001*** ^a^
	**BMRS Independent Variables**						
	Age range	-0,158	0,124	0,205	-0,403	0,087	R2 = 0,134 F= 3,251 ***p = 0,002*** ^a^
	Educational status	-0,052	0,062	0,403	-0,176	0,071	
	Spouse's educational status	-0,162	0,103	0,119	-0,366	0,042	
	Kinship with spouse	-0,068	0,132	0,608	0,330	0,194	
	History of abortion	0,262	0,133	***0,049***	-0,001	0,525	
	History of curettage	0,205	0,149	0,171	-0,090	0,500	
	Previous delivery type	-0,312	0,229	0,175	-0,766	0,141	
	Prior use of family planning method	-0,248	0,130	0,060	-0,506	0,010	
**CESAREAN DELIVERY**	**BSS-R Independent Variables**						
	Educational status	-0,462	0,208	***0,027***	-0,872	-0,053	R2 = 0,045 F=4,763 ***p = 0,003*** ^a^
	Kinship with spouse	1,276	0,495	***0,011***	0,301	2,250	
	History of abortion	1,066	0,576	0,065	-0,069	2,200	
	**DAPS Independent Variables**						
	Kinship with spouse	0,233	0,067	***<,001***	0,101	0,364	R2 = 0,076 F = 7,636 ***p<,001*** ^a^
	Family type	-0,203	0,114	0,077	-0,427	0,022	
	Prior use of family planning method	0,180	0,079	***0,023***	0,025	0,335	
	**BMRS Independent Variables**						
	Educational status	-0,126	0,051	***0,013***	-0,225	-0,026	R2 = 0,075 F = 5,910 ***p<,001*** ^a^
	Spouse's educational status	0,043	0,086	0,615	-0,126	0,213	
	Prior use of family planning method	-0,298	0,107	***0,006***	-0,508	-0,087	
	Aile için planlanılan çocuk sayısı	0,167	0,084	***0,048***	0,002	0,332	

^a^multiple linear regression

## Discussion

The current study reveals significant differences between groups of participants in terms of total scores of the BSS-R according to the mode of delivery. Women who had vaginal deliveries reported higher levels of satisfaction with their birth experiences compared to those who delivered by cesarean section. Additionally, significant differences were found between groups in the BMRS sub-dimensions of “memory centrality” and “memory consistency,” which assess how birth memories are anchored and recalled consistently in the mind. These findings suggest that the mode of delivery may affect not only physical but also psychological and cognitive processes.

There are numerous studies in the literature examining the influence of age on the preference for mode of delivery during pregnancy [17, 18]. Some research indicates that advanced maternal age increases cesarean section rates [19], in contrast, others show that cesarean delivery is more commonly preferred in the 20–29 age group [20]. In the current study, the women’s ages were identified as an important variable affecting the mode of delivery. The majority of participants were young women aged 18–28, and the rate of vaginal delivery in this age group was higher compared to the older age group. This suggests that pregnancy at a younger age may be associated with a tendency towards vaginal birth and that age as a factor is linked not only to biological to but also to socio-cultural preferences.

In this study, it was determined that the experience of the previous mode of delivery influenced the preferred mode of delivery in the current pregnancy. Similarly, the literature reports that having a cesarean section in a previous birth increases the likelihood of cesarean preference in subsequent deliveries [21, 22]. Our findings are consistent with this literature. Notably, the widespread belief that a previous cesarean necessitates cesarean deliveries in subsequent births, along with the very low rates of vaginal birth after cesarean (VBAC) in our country, is an important factor that may explain this result. This indicates that both clinical practices and cultural beliefs play a decisive role in birth preferences.

In the current study, a significant difference was found between the mean total scores of the BSS-R for different modes of delivery (*p* < 0.05). While some studies in the literature report that women who had cesarean deliveries expressed higher satisfaction [23], another study conducted in Turkey found that women who had vaginal deliveries experienced greater satisfaction compared to those who had cesarean Sect. [24]. A study conducted in the Netherlands also revealed that women who had vaginal deliveries had higher satisfaction levels when interviewed by phone two to four weeks postpartum [25]. The higher satisfaction observed among women who underwent cesarean delivery in our study may be related to the decline in midwife-led births and various negative birth experiences encountered by women. Additionally, difficulties in coping with labor pain are thought to influence the preference for cesarean delivery. This situation indicates that birth satisfaction is a multidimensional concept that depends not only on the mode of delivery but also on healthcare service provision and individual experiences.

In our study, a significant relationship was found between BSS-R scores and the educational levels of women and their partners (*p* < 0.005). Notably, among women who had vaginal deliveries, lower educational levels were associated with higher satisfaction levels. While most studies in the literature using the BSS-R report no significant difference between educational level and total scale scores [26, 27], some studies supporting our findings have identified significant differences between women’s educational levels and birth satisfaction [28]. These discrepancies among studies are thought to be due to cultural and regional factors.

In our study, a significant difference was found between spousal consanguinity status and mean BSS-R scores: women who were not related to their spouses exhibited higher birth satisfaction (*p* < 0.005). The literature indicates that women generally make decisions about their mode of delivery themselves, although this decision-making process may also involve their mothers, spouses, close relatives, or mothers-in-law [29]. A study by Şahin et al. (2020) found significant differences in mode of delivery preferences based on spousal consanguinity status [30]. Our findings are consistent with the literature and support the idea that women without spousal consanguinity may have greater autonomy in making their birth decisions, which contributes to increased birth satisfaction.

In our study, no significant difference was found between the total and subscale scores of the DAPS across different modes of delivery (*p* > 0.005). However, the total mean DAPS score of women who had cesarean deliveries (2.33 ± 0.53) was slightly higher than that of women who had vaginal deliveries (2.29 ± 0.61), indicating a tendency among these women to avoid future pregnancies. Supporting our findings, Barut et al. (2022) reported a similar mean total DAPS score of 2.16 ± 1.04 [31]. According to the 2018 Turkey Demographic and Health Survey, the total fertility rate is 2.3, and the cesarean delivery rate has increased to 52% [32]. These data support the idea that rising cesarean rates may trigger pregnancy avoidance and highlight the impact of mode of delivery on pregnancy attitudes.

In our study, a significant difference was found when comparing total DAPS scores by educational status according to the mode of delivery (*P* < 0.005). Women with a university degree or higher had higher DAPS scores than those with lower educational levels. A significant relationship between educational status and DAPS scores was particularly observed among women who had vaginal deliveries. In a study conducted by Barut et al. (2022), significant differences were also reported between women’s educational levels and their perception of childbirth as a medical event [31]. These results can be interpreted as indicating that higher education levels may increase the desire to avoid pregnancy.

In our study, when comparing the total and subscale mean scores of BSS-R by mode of delivery, significant differences were found, particularly in the subscales of Centrality of Memory and Consistency. The total mean score of women who had vaginal deliveries (4.54 ± 0.69) was higher than that of those who had cesarean deliveries (4.48 ± 0.72). According to the literature, negative memories of childbirth can adversely affect the maternal role, and women with such memories are reported to have a higher risk of developing postpartum depression [33, 34]. Additionally, there are findings indicating a positive relationship between maternal functioning and BSS-R total and subscale scores [35]. Higher scores on the BMRS indicate a more negative birth experience. The higher scores observed in vaginal deliveries compared to cesarean deliveries in our study may be attributed to the more intense pain and longer duration of labor associated with vaginal birth.

In our study, a significant difference was found in the mean BSS-R scores according to the educational status based on the mode of delivery (*p* < 0.05). As the level of education decreased, the intensity and frequency of birth memory recollection increased. Similarly, in a study by Sarısoy and Tuğut, women with primary education had higher mean BSS-R scores compared to university graduates [36]. These findings highlight the effect of educational level on the perception of birth experience and are consistent with the literature.

### Strengths and limitations of study

This study has several strengths. The sample size was determined as a result of the power analysis performed beforehand with the G*Power program, and thus sufficient statistical power was achieved. Working with a sample that included equal numbers of women who gave birth vaginally and by cesarean section allowed for balanced and meaningful comparisons based on mode of delivery. In addition, collecting the data at two different times, at 12 h and 7 days postpartum, contributed to the evaluation of both early satisfaction with childbirth and short-term birth memory. The validity and reliability of all the measurement tools used have been previously proven, and Cronbach’s alpha values were at an acceptable level in this study, which increased the reliability of the results.

This study was conducted only with women hospitalized in the postpartum ward of a university hospital in the Central Black Sea region, which limits the generalizability of the findings to women giving birth in different regions, or in different health institutions.

## Conclusion

This study identified a significant difference between the mode of delivery and childbirth satisfaction, while no significant differences were found in pregnancy avoidance, birth memory, or recollection (*p* < 0.05). Based on these findings, it is essential to ensure that women have positive experiences during the antenatal, birth, and postpartum periods. These results highlight the importance of midwives providing necessary information and counseling to women and their families about labor and delivery options as part of antenatal care services. Additionally, the higher satisfaction rate among women who had cesarean deliveries in this study underscores the need for interventions aimed at improving satisfaction with vaginal deliveries. Reducing cesarean delivery rates and encouraging vaginal births, are crucial both for women’s health and for mitigating the economic burden on the healthcare system. Ensuring that women make informed decisions about their mode of delivery requires access to high-quality, comprehensive care from the preconception period through the postpartum phase. This care should include effective counseling on alternative birthing methods, pain management strategies during labor, and relaxation techniques, all of which contribute to a positive childbirth experience.

## Data Availability

The data of this research can be used by contacting and requesting the authors.
